# Ex Pede Herculem

**Published:** 1885-08

**Authors:** 


### EX PEDE HERCULEM.

That was an exceedingly dull person, says DR. HOLMES, who made the remark, *Ex pede Herculem.* He might as well have said, "From a peck of apples you may judge of the barrel." We are told that CUVIER constructed a megatherium from a tooth, that LEIDY and AGASSIZ drew portraits of undiscovered fishes from scales. An interesting psychological problem is presented to us by an editorial, entitled, THE RUMP CONGRESS OF 1887, appearing in *The New York Medical Journal,* of 11th July, 1885, and containing statements, which deserve to be recorded, as revealing the animus of the *Journal's* action with reference to the NINTH INTERNATIONAL MEDICAL CONGRESS. An extract of the editorial reads as follows:

"And all this disgrace is the logical outcome of the false and artificial issues which for the past three years have enabled men in no way representative of the profession to masquerade as its leaders, through the medium of that degenerate and utterly ridiculous concern, the American Medical Association. That organization long ago ceased to work for the benefit of the profession, and for a number of years past its annual meetings have been little more than sources of the most shameless intrigue and demagogism."

It is the old story of "No case; abuse the plaintiff's attorney."

Of the actual facts involved in the case, our esteemed contemporary ought to be fully cognizant. If he is not, his attention is invited to an editorial, appearing in *The Journal of the American Medical Association*, 25th July, 1885, which states three important truths, which must be clearly and distinctly borne in mind.

"(*a.*) That the American Medical Association is the only organized representative of all departments of the profession in the United States; (*b*) that the Committee of Invitation with all its powers and duties was simply the instrument or agent appointed by the Association to perform certain acts or duties; and (*c*) having appointed such agent and invested it with certain important powers, the Association was, by the fundamental principles of parliamentary law as well as by the dictates of common sense, itself responsible to the medical world, both for the character of the agent or committee, and the manner in which it should discharge its duties."

Possibly, while our valued *confrère* has been in possession of the actual facts involved in the case, he has been unable to arrive at a logical conclusion.

In such event, it is suggested that the study of PROFESSOR JEVONS'S excellent little treatise on "*The Necessary Laws of Thought*" might be attended with benefit to himself, and advantage to his readers. It seems probable, however, that the pitiful begging of the question upon the part of the *New York Medical Journal* is not due to ignorance of the facts involved, nor to the incorrect application of logical processes.

In seeking a solution of the problem, may not the following words of a great physician, and a shrewd observer of men, throw some light on the subject:

"A mixture of a life doth ever add pleasure. Doth any man doubt, that if there were taken out of men's minds vain opinions, flattering hopes, false valuations, imaginations as one would, and the like, but it would leave the minds of a number of men poor shrunken things, full of melancholy and indisposition, and unpleasing to themselves."

Whatever the motive of our journalistic brother may have been,—whether ignorance of the facts involved in the case, defective knowledge of logical processes, or that unfortunate moral obliquity, consisting in incorrespondence of the subjective with the objective order of the phenomena,—his temerity, in deliberately offering insult to every member of the American Medical Association excites surprise.

As a commercial venture,—and we are compelled by the *Journal's* open advocacy of the propriety of consultation with irregular practitioners, that considerations of pecuniary return constitute the basis of professional ethics,—it must prove a failure. Neither will the imposing publishing house of Bond street, nor its exponent, the *Journal*, reap material reward. Surely, every member of the American Medical Association will bear this sentence in mind, in the future, remembering the words of O. W. Holmes, that "As the 'O' revealed Giotto,—as the one word '*moi*' betrayed the Stratford-atte-Bowe-taught Anglais,—so all a man's antecedents and possibilities are summed up in a single utterance which gives at once the gauge of his education and his mental organization."

